# Genetic variability of the *Aedes aegypti* (Diptera: Culicidae) mosquito in El Salvador, vector of dengue, yellow fever, chikungunya and Zika

**DOI:** 10.1186/s13071-018-3226-5

**Published:** 2018-12-14

**Authors:** Andrea L. Joyce, Melany Murillo Torres, Ryan Torres, Miguel Moreno

**Affiliations:** 1 Public Health, University of California, 5200 North Lake Road, Merced, CA 95343 USA; 2 Departmento de Biología, Universidad de El Salvador, Final de Av. Mártires y Héroes del 30 Julio, San Salvador, El Salvador

**Keywords:** AFLPs, Mitochondrial DNA *cox*1, Dengue, Chikungunya, Yellow fever, Zika, *Aedes aegypti*, Central America, Barcode, Haplotype, Eradication

## Abstract

**Background:**

*Aedes aegypti* is associated with dengue, yellow fever, chikungunya and Zika viruses. This vector is widespread in tropical and subtropical areas, and can also occur in temperate areas at higher latitudes. The geographical distribution of *Ae. aegypti* continues to spread due to human activities. This is the first study to examine the population genetic structure of this insect in El Salvador, Central America.

**Methods:**

*Aedes aegypti* larvae were collected from six geographical regions of El Salvador: Sonsonate, San Salvador, Chalatenango, Usulután, San Miguel and Morazán. Larvae were raised into adults, identified and preserved. Two molecular markers, amplified fragment length polymorphism (AFLP) genotyping and mitochondrial DNA (mtDNA) cytochrome *c* oxidase subunit 1 (*cox*1) sequencing, were used to investigate population genetic structure.

**Results:**

Structure analysis found two genetically distinct populations; one occurs predominantly in the north and west, and a mix of two populations occurs in the southeast of the country. Genetic distances ranged from 0.028 (2.8%) to 0.091 (9%), and an AMOVA analysis found 11% variation between populations. Mitochondrial DNA *cox*1 sequences produced a haplotype network which consisted of 3 haplogroups and 10 haplotypes. Haplogroup 1 had low haplotype and nucleotide diversity and was found in all six regions. Haplogroups 2 and 3 had higher haplotype and nucleotide diversity, and were less abundant; haplogroup 3 was found in only 3 of the six regions studied. Bottleneck tests were significant, suggesting that populations had undergone a recent bottleneck. A maximum likelihood tree, which combined samples from this study with available sequences in GenBank, suggested that two genetically divergent lineages had been introduced.

**Conclusions:**

Relatively high genetic diversity was found in *Ae. aegypti* in El Salvador. The mtDNA sequences clustered into two lineages, as found in previous studies. Samples in El Salvador may be introduced from regions in North and South America where past eradication was not complete. Future study of genotypes in surrounding countries would provide a more complete picture of the movement and potential source of introductions of this vector. The distribution of the lineages and haplogroups may further our understanding of the epidemiology of *Ae. aegypti* associated vector borne diseases.

**Electronic supplementary material:**

The online version of this article (10.1186/s13071-018-3226-5) contains supplementary material, which is available to authorized users.

## Background

The mosquito *Aedes aegypti* is associated with yellow fever, dengue, chikungunya and Zika viruses. These diseases impact the health of millions globally each year. It is estimated that at least 50 million per year are infected with dengue, with inhabitants of 100 or more countries at risk of infection [1]. *Aedes aegypti* typically occurs in tropical and subtropical regions of the world, but can also occur in temperate regions at higher latitudes [2]. This mosquito continues to be transported around the globe with human movement and global commerce, exerting a major impact on public health.

Studies have examined genetic diversity of *Ae. aegypti* on a global scale [3–7]. In Africa, the region of origin, two forms of *Ae. aegypti* vary in their ecology and are considered subspecies. The sylvatic ancestral form of *Ae. aegypti*, *Ae. aegypti formosus*, resides primarily in forested environments and tree holes, and a second anthropophilic form, *Ae. aegypti aegypti*, occurs in urban areas in homes where water is stored [3, 7–9]. The evolutionary history of the vector is complex, yet studies suggest that mosquitoes have been moved from Africa to America and then from America to Asia, an idea supported by the finding that populations in America have higher genetic diversity than those in Asia [2, 5]. In addition, evidence suggests that different lineages have been transported out of East and West Africa [6, 10].

Regional studies have investigated the genetic diversity of introduced *Ae. aegypti* in numerous countries in the Western Hemisphere, Asia and Australia. A variety of molecular markers have been utilized in these studies including microsatellites and mitochondrial DNA. Studies have found evidence for one, two or three introduced clades of *Ae. aegypti* outside of their region of origin of Africa [11–13]. For example, collections of *Ae. aegypti* from the northeast coast of Mexico suggest two divergent lineages and two introductions [14]. In Brazil, evidence was found for at least two introduced lineages [15, 16]. Results from Argentina suggested three lineages of *Ae. aegypti*; low nucleotide diversity suggested passive dispersal between Argentine populations and those from adjacent countries [12]. In Bolivia, two divergent populations were found with low nucleotide diversity, suggesting a recent introduction or a small founding population [13]. Another source of population divergence in *Ae. aegypti* in the Western Hemisphere could be populations that persisted through eradication programs, which might reinvade surrounding regions [16–18]. In Brazil, population genetic studies suggested that after eradication, *Ae. aegypti* was later reintroduced [16]. In northern Brazil, *Ae. aegypti* were more closely related to those from Venezuela, where eradication did not occur. The former study along with a subsequent study in Brazil suggested that the *Ae. aegypti* populations currently found in Brazil are populations which were reintroduced in the country after eradication was achieved [16, 17]. Other studies found that once *Ae. aegypti* are introduced into a region, they could undergo genetic divergence due to environmental factors and geographical barriers [19, 20]. For example, populations of *Ae. aegypti* in Peru were found to be divergent on different sides of the Andes, possibly due to climatic differences and geographical isolation [19, 20].

In El Salvador, Central America, *Ae. aegypti* is the primary vector of dengue, chikungunya and Zika virus [21]. El Salvador is a relatively small country, with a Pacific coast and varied topography and climate. *Aedes aegypti* was previously eradicated from El Salvador, and from much of the Western Hemisphere, in the 1950s and 1960s [18, 22]. Similarly, in the countries surrounding El Salvador, eradication was also achieved: in Nicaragua by 1958, and in Guatemala and Honduras by 1959. El Salvador was declared eradicated by 1960, and Costa Rica was free of *Ae. aegypti* by 1961. However, in El Salvador, *Ae. aegypti* was reintroduced in a shipment of tires from the USA to San Salvador in 1965 [23]. By 1982, an epidemic of dengue was reported, and in 1983, 2867 cases were reported from throughout the country [24, 25]. The mosquito may additionally have been reintroduced through ports, human movement, or along transportation routes from nearby countries, or small pockets of mosquitoes may have survived in isolated refugia. *Aedes aegypti* is currently widespread through the country. In 2014, chikungunya cases were first reported in El Salvador, followed by the first reports of Zika in 2015 [21]. Previous studies have suggested that knowledge of the genetic variability of a vector could contribute to vector control and reducing cases of vector-borne disease [26]. For example, strains of *Ae. aegypti* can vary in vector competence and insecticide resistance, and thus respond differentially to control [26]. Little is known about the population genetic structure of *Ae. aegypti* in Central America. The objectives of this study were to examine the genetic variability of populations of *Ae. aegypti* in six regions of El Salvador, and to investigate the number of possible introductions and lineages of this insect in the country.

## Methods

### Study sites

**Fig. 1:**
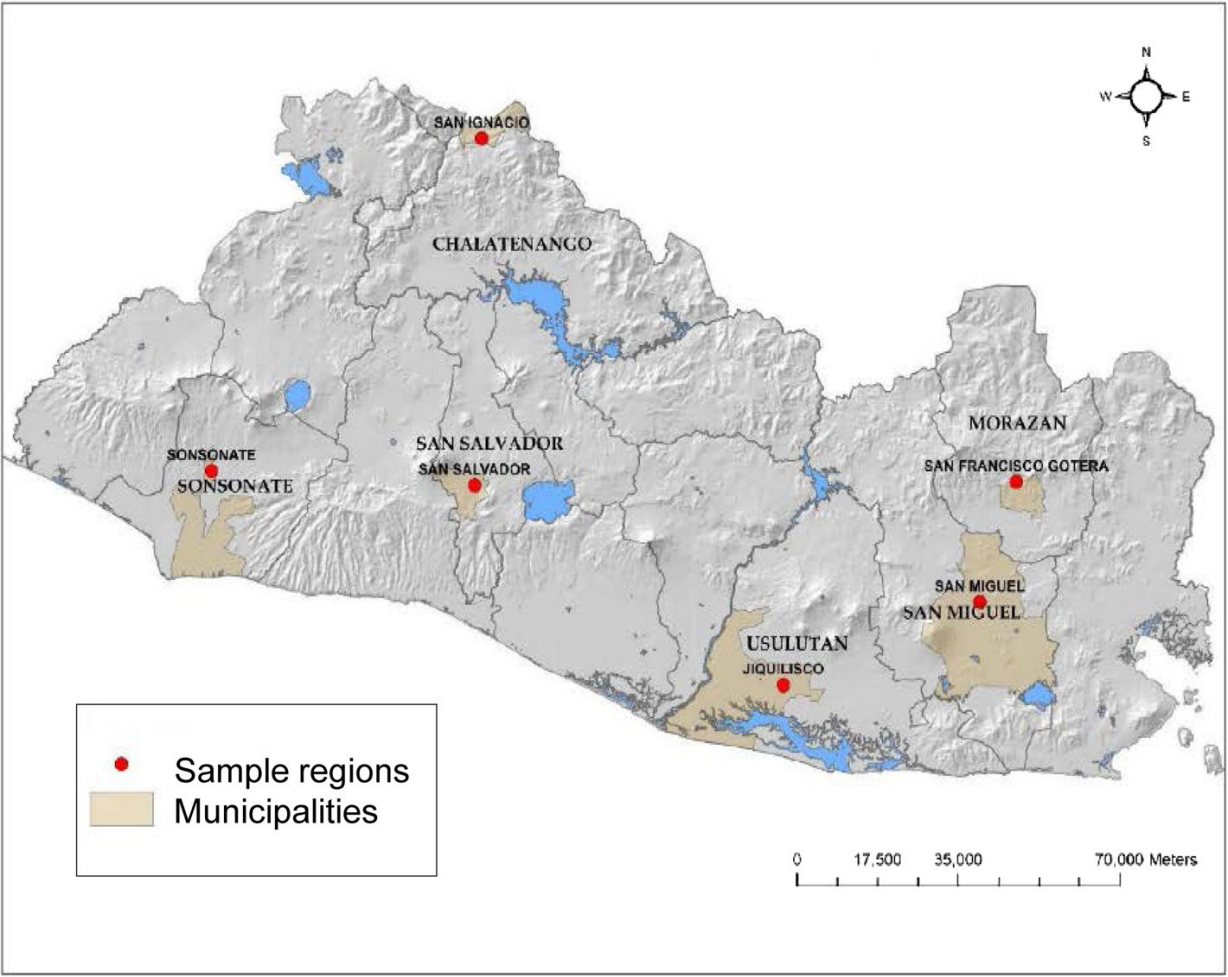
Collection locations including the municipality and department where samples of *Ae. aegypti* were obtained in El Salvador

The study was conducted between May-August 2014 in El Salvador during the rainy season. Larvae of *Ae. aegypti* were collected in six departments of the country: Sonsonate, San Salvador, Chalatenango, Usulután, San Miguel and Morazán (Fig. 1). These six departments were located in three different regions of El Salvador. The western zone of the country (Sonsonate) has a port on the Pacific coast in Acajutla. The central zone consists of San Salvador and Chalatenango; San Salvador is the capital and Chalatenango is a sparsely populated mountainous region which borders Honduras. In the south and eastern parts of the country, collections were made in Usulután, San Miguel and Morazán (Fig. 1). Usulután borders the Pacific coast and includes the Bay of Jiquilisco and nature reserves. San Miguel is a hot, dry interior region of the country, located on the Pan American Highway. Finally, Morazán is a department located between San Miguel and the border of Honduras (Fig. 1). In each department, one or two neighborhoods were visited to collect samples, with at least two or three sites per neighborhood used to collect larvae (Table 1). For example, the samples collected in Sonsonate were from two neighborhoods, San Antonio and El Carmen, with three sampling sites within each neighborhood (Table 1). Sample sites within each neighborhood included barrels of standing water and outdoor sinks, commonly called ‘pilas’. The samples collected from each site in this study typically consisted of a few of the numerous larvae which were present in a pila or barrel. In addition, findings of a previous study found that the mean number of families represented per oviposition site for *Ae. aegypti* was 4.7 [27]. This previous research, along with the sampling design in the present study, reduced the chance that samples collected were siblings. After collection, larvae were placed in a glass jar for transport to the laboratory for rearing at the University of El Salvador.

**Table 1:** Collection sites for *Ae. aegypti* used in this study

Department	Municipality	Neighborhood	Sample codes	Collection date (2014)	Latitude	Longitude
Sonsonate	Sonsonate	El Carmen	SON 1–5	Aug 18	13°43'40.58"N	-89°44'1.40"E
			SON 6–10		13°43'42.55"N	-89°44'6.11"E
			SON 11–14		13°43'43.17"N	-89°44'6.94"E
		San Antonio	SON 15–19	Aug 18	13°43'40.11"N	-89°44'5.17"E
			SON 20–22		13°43'38.62"N	-89°44'4.89"E
			SON 23–25		13°43'36.17"N	-89°44'6.39"E
San Salvador	San Salvador	La Fosa	SS 1–3	May 9	13°43'14.42"N	-89°11'55.67"E
			SS 4–5		13°43'14.70"N	-89°11'56.97"E
			SS 6–9		13°43'14.87"N	-89°11'55.42"E
			SS 10–11, 13		13°43'13.69"N	-89°11'56.24"E
			SS 14–16		13°43'14.03"N	-89°11'52.76"E
			SS 17–19		13°43'11.65"N	-89°11'55.52"E
		San Jacinto	SS 20–22	May 16	13°41'3.32"N	-89°10'48.41"E
			SS 23		13°41'5.44"N	-89°10'46.94"E
Chalatenango	San Ignacio	La Villa	SI 1–2, 4–5	Aug 29	14°20'21.11"N	-89°10'44.58"E
			SI 6 8–10		14°20'19.69"N	-89°10'44.58"E
			SI 11–13		14°20'19.01"N	-89°10'43.75"E
			SI 15–18		14°20'22.01"N	-89°10'42.58"E
			SI 19–23		14°20'17.69"N	-89°10'40.87"E
			SI 23–24	Aug 29	14°20'18.52"N	-89°10'39.35"E
Usulutan	Jiquilisco	Las Flores Siembras	JC 1	June 5	13°19'33.88"N	-88°34'18.50"E
			JC 4–6		13°19'32.11"N	-88°34'16.48"E
			JC 7–10		13°20'8.87"N	-88°34'21.86"E
			JC 11–13		13°20'11.69"N	-88°34'22.42"E
			JC 15–17		13°19'37.89"N	-88°34'12.80"E
		Las Flores	JC 18–21		13°19'37.51"N	-88°34'6.99"E
			JC 22–24		13°19'37.54"N	-88°34'6.96"E
San Miguel	San Miguel	Las Americas	SM 1–4	June 5	13°28'41.55"N	-88°10'19.34"E
			SM 5–12		13°28'40.85"N	-88°10'22.68"E
			SM 13–17		13°28'42.19"N	-88°10'22.82"E
			SM 18–23		13°28'40.76"N	-88°10'20.14"E
			SM 24		13°28'43.33"N	-88°10'18.23"E
Morazan	San Francisco de Gotera	Morazan	MZ 1–3	July 27	13°41'7.87"N	-88° 5'54.10"E
			MZ 4–10		13°41'7.30"N	-88° 5'53.84"E
			MZ 11–14		13°41'8.19"N	-88° 5'56.27"E
			MZ 15–17		13°40'54.08"N	-88° 5'59.03"E
			MZ 18		13°40'54.51"N	-88° 5'58.76"E
			MZ 20–22		13°40'54.84"N	-88° 6'0.29"E
			MZ 25		13°40'52.35"N	-88° 6'3.14"E
			MZ 27, 29		13°40'51.41"N	-88° 6'3.80"E

### Rearing larvae and adult identification

Larvae were transported to the Center for Health Research (CENSALUD) at the University of El Salvador, in San Salvador. In the laboratory, larvae from each collection site were placed into plastic cups with distilled water and maintained in wire mesh cages (20 × 10 × 10 cm) until adults emerged. Each day, cages were checked for newly emerged adults, which were frozen and later identified to the species level based on morphology. Only adult females were used for this study.

### DNA extraction

Adult females were used for DNA extraction, with at least 30 adult females individually extracted from each of the six regions. DNA extraction was completed with the Qiagen DNEasy® Blood and Tissue Extraction Kit (Qiagen, Venlo, Netherlands) following standard protocols [28], using an overnight incubation of the samples at 65 °C. The quantity of DNA in each sample was measured using the Qubit 2.0 fluorimeter Hs DNA kit (ThermoFisher, Waltham, MA, USA) and averaged 50 ng/μl.

### Population genetic structure: amplified fragment length polymorphisms (AFLPs)

Amplified fragment length polymorphisms were produced for each mosquito using three primer combinations with procedures described by [29] and modified by Joyce et al. [30]. The three primer combinations used were the following: (i) *Mse*I-CAT/*Eco*RI-ACG; (ii) *Mse*I-CAC/*Eco*RI-ACT; and (iii) *Mse*I-CAC/*Eco*RI-ACA. Details of AFLP reactions are described in Joyce et al. [30]. Initial DNA template for reactions used 5 μl (~200 ng DNA). Prior to capillary electrophoresis, 0.4 μl of GeneScan Liz 500 size standard and 0.9 μl of HiDi formamide (ThermoFisher, Waltham, MA, USA) were added to 1 μl of the final product of each sample. Samples were run on an Applied Biosystems 3730 Genetic Analyzer (Thermo Fisher, Waltham, MA, USA). Genemapper 5.0 software was used to determine the presence or absence of each allele. The peak detection threshold was set for each primer combination and was typically 100 luminescent units. Phylip 3.695 was used to calculate Nei’s pairwise genetic distance and to generate a neighbor-joining tree used to visualize genetic similarity of individuals. Structure 2.3.4 [31] was run using the following parameters: a ‘burn-in’ of 50,000 iterations, followed by 50,000 iterations, an admixture model, and independent loci. The maximum number of potential populations for K was set as the number of geographic sampling locations plus 4 (K = 6 locations + 4 = 10) as suggested by Pritchard et al. [32], and each iteration was run 20 times. The Structure output was used as input for Structure Harvester [33] using the method of Evanno et al. [34] to determine the most likely value for K. Clumpak software was used to permutate runs of K = 2, with Distruct was used visualize results [35].

A Mantel test was used to examine the relationship between genetic distance and geographical distance (isolation by distance) using GenAlex 6.5 [36]. An analysis of molecular variation was run to examine genetic variation among the six regions, using GenAlex 6.5 and 999 permutations. A principal components analysis was also performed with GenAlex 6.5.

Bottleneck version 1.2.02 was used to test whether there was a significant departure from an equilibrium expectation of heterozygotes in each population [37]. We used polymorphic loci from the AFLP data for each population in the six regions, and for individuals in each of the three haplogroups. The infinite allele model (IAM) was run for 1000 iterations, and results were examined with the Wilcoxon sign rank test.

### Mitochondrial DNA *cox*1

For each insect, DNA was used to sequence a ~650 bp region of mitochondrial DNA cytochrome *c* oxidase subunit 1 (*cox*1) (known as the ‘barcode’) using the universal forward primer LCO1490 (5'-GGT CAA CAA ATC ATA AAG ATA TTG G-3') and the reverse primer HCO2198 (5'-TAA ACT TCA GGG TGA CCA AAA AAT CA-3') [38, 39]. A polymerase chain reaction (PCR) mix for six samples consisted of the following: 195.6 μl sterile ultra-pure water; 2.4 μl Taq polymerase (Clonetech, Mountainview, CA, USA); 30 μl Taq 10× buffer; 24 μl dNTPs; 6 μl forward primer; and 6 μl reverse primer and 2 μl template DNA (~100 ng template). For each reaction, 2 μl template DNA was added to each vial and the contents were vortexed and spun down. The PCR program was as follows: an initial 1 min warm-up at 95 °C; then 40 cycles of a touchdown program consisting of 92 °C for 30 s, 43–52 °C for 30 s (with a 0.3 °C temperature increase each s), and 72 °C for 60 s; after 40 cycles, a 68 °C final extension for 10 min and then a hold at 4 °C. PCR products were run on a 1.5% agarose gel to visualize the amplification of products of ~650 bp. Samples were cleaned-up using the Exo-sap-it (Affymetrix, Inc, Santa Clara, CA, USA) cleanup kit and run on a 3730 Genetic Analyzer. Resulting sequences were analyzed using Geneious 7 (Biomatters, Aukland, New Zealand) software to produce consensus sequences [40]. Sequences were trimmed, forward and reverse sequences were aligned, and a consensus sequence was produced. Sequences were aligned in Geneious 7.0 using the Clustal W alignment function and used to produce an unrooted neighbor-joining (NJ) tree. We also generated a phylogenetic tree using model-based maximum likelihood (ML) analysis for the same dataset [41]. Using the model selection option in MEGA 7.0, we found that the Tamura 3 parameter with Gamma distribution (G) was the best-fit model to our dataset based on the lowest BIC (Bayesian Information Criterion) value [41]. ML analysis used this best-fit model and clade support was assessed *via* 1000 bootstrap replicates [41].

The mitochondrial DNA *cox*1 sequences were used to determine the overall number of haplotypes, haplotype diversity, nucleotide diversity and Tajima’s D using DNAsp 5.10 [42]; Tajima’s D was calculated to test whether there was a departure from neutrality, such as a population expansion or contraction. Subsequently, these same parameters were determined for each of the six regions of the country, and for the mitochondrial DNA haplogroups. Results were used to construct a haplotype network using PopArt 1.7 and selecting the TCS option [43].

Additionally, a combined maximum likelihood phylogenetic tree was constructed using samples from El Salvador and individuals of *Ae. aegypti* previously sequenced which were available in the GenBank database. Samples included in the tree were selected in the following manner. First, one individual from each of the three most common mitochondrial haplogroups in El Salvador was used for a blast search. Resulting sequences which were 99–100% similar were retrieved, and those from North and South America were selected for inclusion in the tree to compare with those from El Salvador [44]. Several samples from East and West Africa were also included for comparison [45]. In addition, several GenBank accessions from Bennett et al. [6] were included, as they were known to be free of nuclear mitochondrial DNA (NUMTs). Concerns have been raised about whether nuclear mitochondrial DNA (NUMTs) may contribute to the presence of multiple lineages when using mitochondrial DNA sequences for phylogenetic studies of *Ae. aegypti* [46]. In this study, we attempted to overcome this issue by including sequences from GenBank in the combined phylogenetic tree from previous studies where samples were known to be free of NUMTs [6]. From El Salvador, at least two individuals from each of the three common haplogroups, and at least 2 individuals from each of the 6 regions were included in the tree. This resulted in 14 samples from El Salvador and 16 sequences from GenBank for the combined phylogenetic analysis. Trees were constructed in a manner similar to those described previously. Sequences were aligned in Geneious 7.0 using the Clustal W alignment function, and a phylogenetic tree was produced using model-based maximum likelihood (ML) analysis [41]. The maximum likelihood tree used the Hasegawa-Kishino-Yano model (H-K-Y) with Gamma distribution with invariant sites (G + I) and clade support was assessed *via* 1000 bootstrap replicates.

## Results

### Amplified fragment length polymorphisms (AFLPs)

**Fig. 2:**
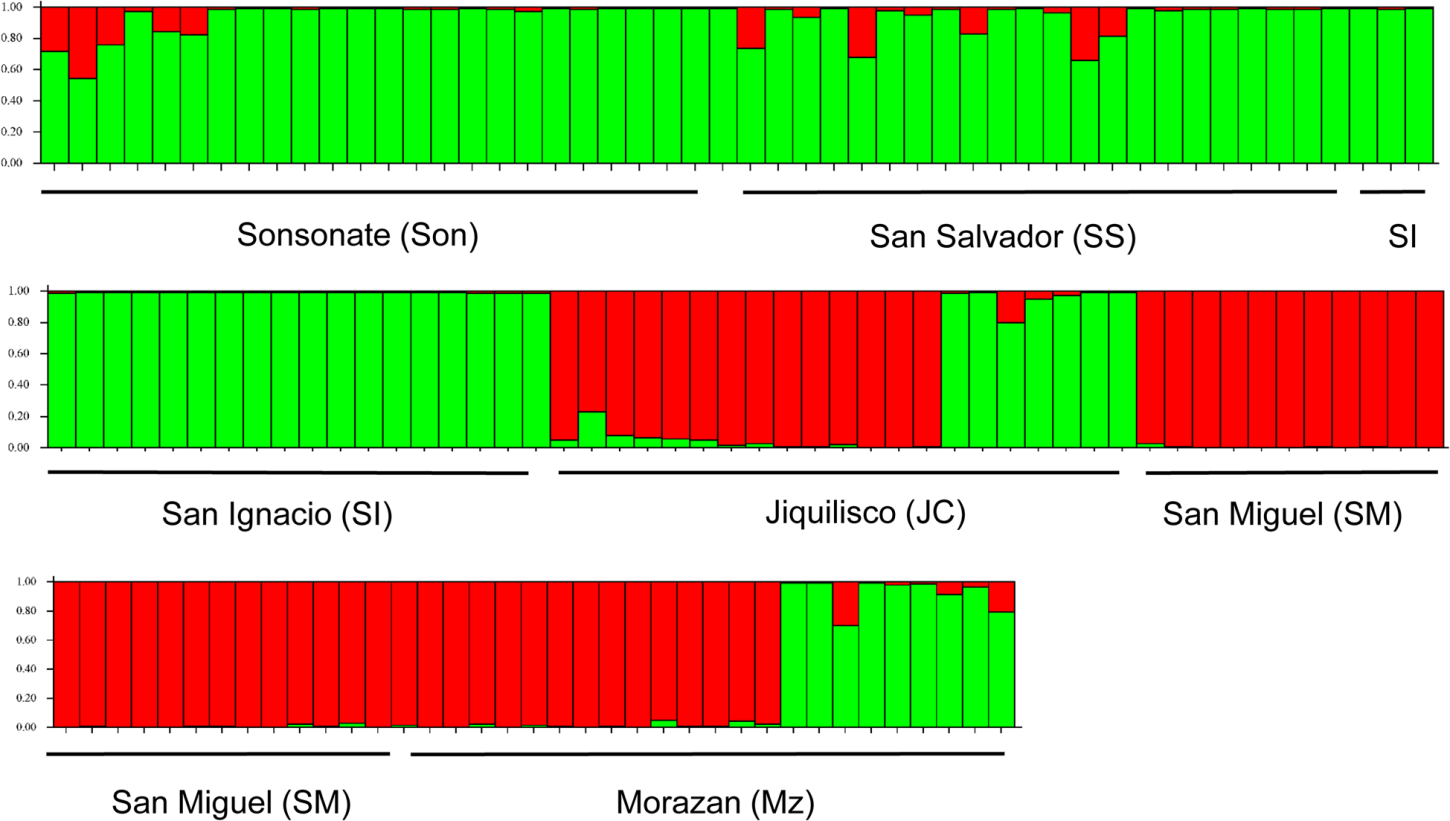
Analysis of population structure for *Ae. aegypti* with the software Structure 2.2.3. Parameters selected were for diploid individuals, 50,000 iterations, admixed data, and independent loci. Each vertical bar represents an individual mosquito. The y-axis shows the probability of an individual being assigned to one of the two genetic clusters. Structure Harvester found that K = 2; there were two genetically distinct populations

From collections in the six regions, 137 samples were used for DNA extraction (Tables 1 and 2). The three AFLP primer combinations used produced 90, 138 and 97 alleles. Structure analysis and subsequent Structure Harvester analysis using the Evanno method found that K = 2 as the mostly likely number of genetically distinct populations (Fig. 2, Additional file 1: Figure S1, Additional file 2: Figure S2). The first group shown in green (Fig. 2) consisted of the individuals from Sonsonate, San Salvador and Chalatenango, the departments located in the northwestern and central part of the country. Samples from the south and east of the country from two departments, Usulután and Morazán, had individuals that clustered into two separate groups (green and red, Fig. 2), while all individuals from San Miguel clustered in the red group (Fig. 2).

**Table 2:** Number of individuals used from each department for amplified fragment length polymorphisms (AFLPs)

No. of individuals	Department	City (Municipality)
25	Sonsonate	Sonsonate
22	San Salvador	San Salvador
21	Chalatenango	San Ignacio
21	Usulután	Jiquilisco
24	San Miguel	San Miguel
24	Morazán	San Francisco de Gotera

Nei’s genetic distance between the populations ranged from 0.028 to 0.091 (Table 3). Smaller genetic distances were found between Morazán and Chalatenango (0.028), Morazán and Jiquilisco (0.028), Sonsonate and San Salvador (0.032), and San Miguel and Jiquilisco (0.035) (Table 3, Fig. 2). The largest genetic distance was between San Miguel and San Ignacio (Chalatenango) (0.091) followed by that between San Miguel and Sonsonate (0.067) (Table 3). The Mantel test to examine the relationship between genetic distance and geographical distance was significant (*r* = 0.976, *P* = 0.010) (Additional file 3: Figure S3). The AMOVA analysis was significant (*P* = 0.001), and found 11% genetic variation among the six populations (Table 4). Bottleneck analyses for individuals from each region, and from the three mtDNA haplogroups (below) found a significant excess of heterozygotes (*P* < 0.001), indicating each population had undergone a recent bottleneck. Results from the IAM model were significant for each of these populations at *P* < 0.001.

**Table 3:** Nei’s genetic distance among populations. Collection locations of all populations are detailed in Table 1

	Son	SS	SI	JC	SM	MZ
Sonsonate	–	0.032	0.054	0.044	0.067	0.059
San Salvador		–	0.048	0.036	0.059	0.053
San Ignacio			–	0.052	0.091	0.028
Jiquilisco				–	0.035	0.028
San Miguel					–	0.044
Morazán						–

*Abbreviations*: *Son* Sonsonate, *SS* San Salvador, *SI* San Ignacio, *JC* Jiquilisco, *SM* San Miguel, *MZ* Morazan

**Table 4:** Results of an analysis of molecular variation (AMOVA) test among six populations

Source^a^	*df*	Sum of squares	Variation (%)	*P*
Among populations	5	723.449	11	0.001
Within populations	131	4934.245	89	
Total	136	5657.693		

*Abbreviation*: *df*, degrees of freedom

^a^*Aedes aegypti* populations from six municipalities in El Salvador, Central America. Collection information is detailed in Table 1

The principal components analysis found that the first axis accounted for 44.75% of the variation, while the second and third axes explained 26.20% and 13.43% of the variation, respectively (Additional file 4: Figure S4). Examining axis 1 (the x-axis) from left to right, there was a separation of the populations from the northwest to the southeast part of the country; samples from Chalatenango, San Salvador and Sonsonate were on the left side of axis 1 (x-axis), while those from Jiquilisco, Morazán and San Miguel (which are in the southeast) located on the right side of axis 1. The left of the second axis (y-axis) separated inland mountainous Chalatenango from coastal Sonsonate and nearby San Salvador, while the top right quadrant (y-axis) had the three populations found in the southeastern portion of the country; Morazán and Jiquilisco clustered together, with San Miguel located at the edge of the same cluster. The six populations clustered into three groups.

### Mitochondrial DNA *cox*1

**Fig. 3:**
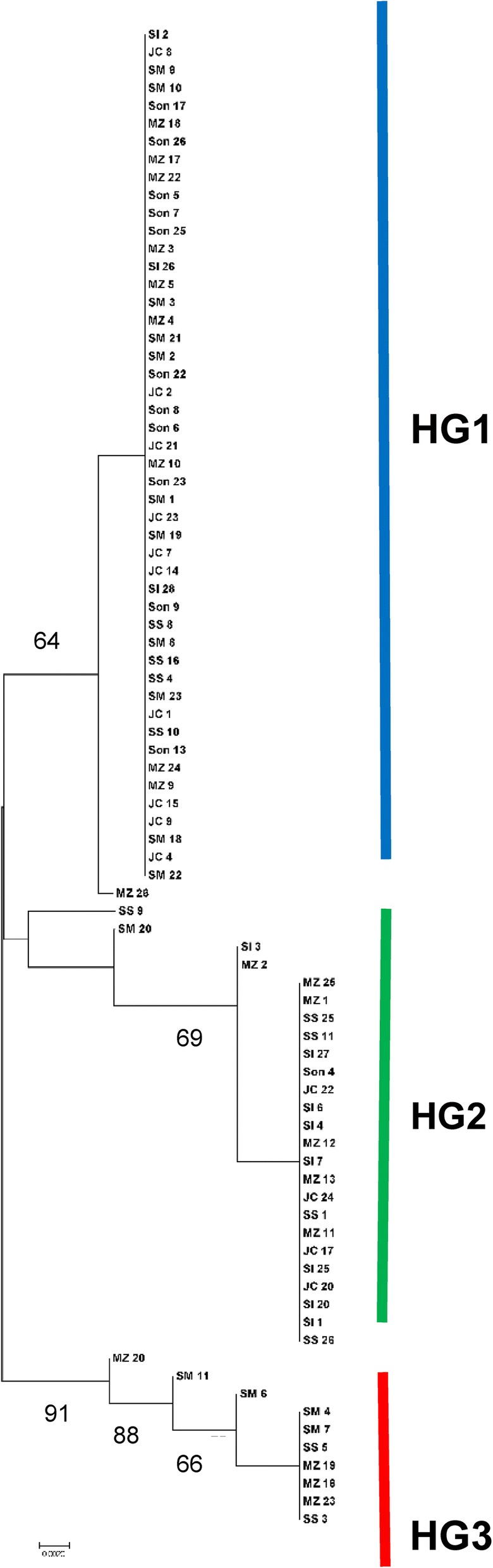
Mitochondrial DNA *cox*1 sequences from 82 *Ae. aegypti* collected in El Salvador. Maximum likekihood tree, Tamura 3 model, 1000 replicates were run and nodes with support above 65% are indicated. Haplogroup 1 (HG1) and haplogroup 2 (HG2): individuals from all six departments; haplogroup 3 (HG3): collections from San Salvador, San Miguel and Morazán. *Abbreviations*: Son, Sonsonate; SS, San Salvador; SI, San Ignacio, Chalatenango; JC, Jiquilisco, Usulután; SM, San Miguel; Mz, Morazán

In total 84 individuals were sequenced from the six regions, with numbers sequenced: Sonsonate (*n* = 12); San Salvador (*n* = 11); San Ignacio (*n* = 11); Jiquilisco (*n* = 14); San Miguel (*n* = 16); and Morazán (*n* = 20). The neighbor-joining consensus tree of individuals from El Salvador produced 3 groups, with 0.01 (1%) genetic distance between groups 1 and 2, and 1% between groups 2 and 3. Between groups 1 and 3, the genetic distance was ~2%. A maximum likelihood tree also indicated three groups (Fig. 3). The largest group in the tree consisted of 49 individuals from all six departments, while the second largest group consisted of 23 individuals from all six departments. Finally, the third main group of the tree consisted of 10 individuals from San Salvador, San Miguel and Morazán (Fig. 3).

**Fig. 4:**
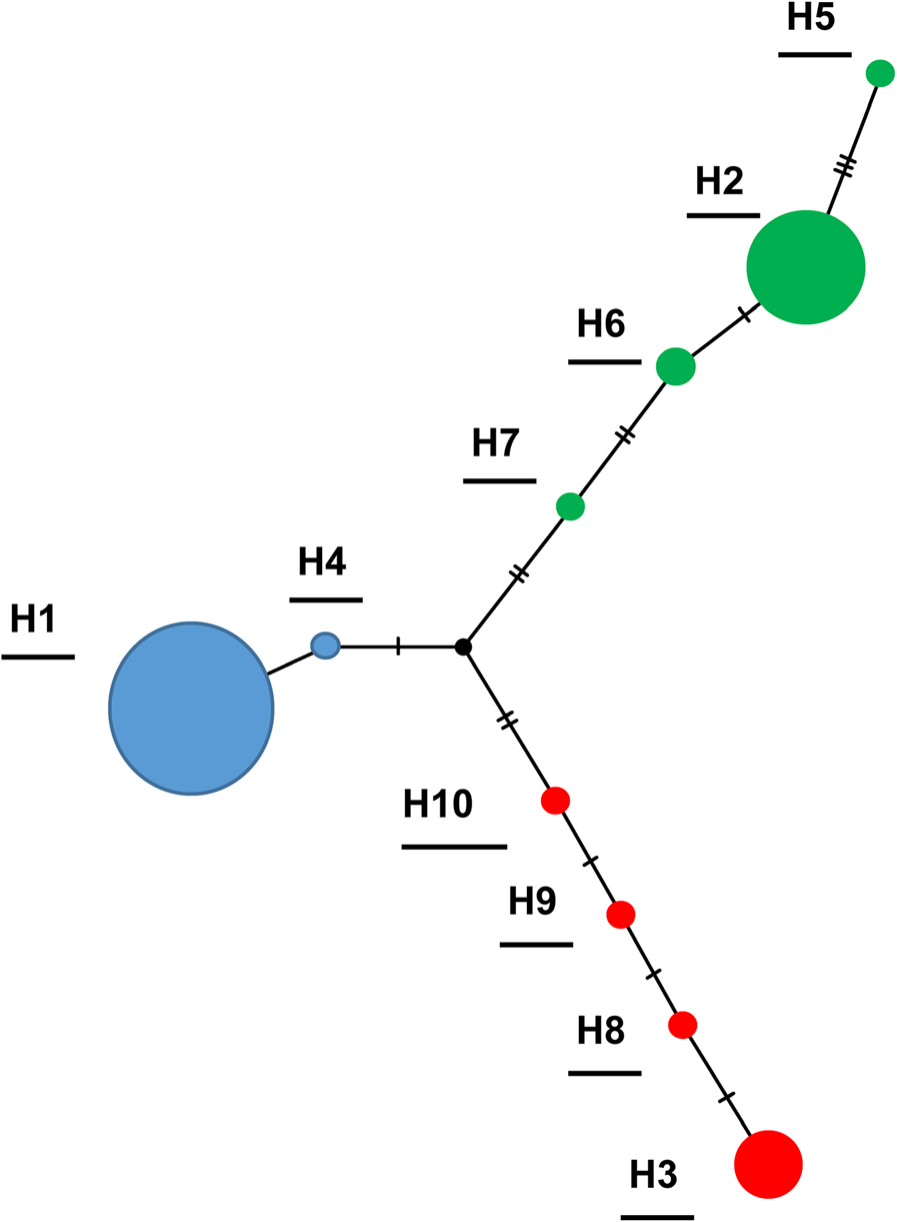
Haplotype network based on 84 mitochondrial DNA *cox*1 sequences of *Ae. aegypti* collected in six regions of El Salvador. Ten haplotypes were found in three haplogroups with an overall haplotype diversity of 0.610

A haplotype network was also constructed using the 84 mtDNA sequences, and was found to consist of 3 branches (Fig. 4), which included a total of 10 haplotypes. The haplotypes are indicated on the haplotype network and three haplogroups are indicated in blue (HG1), green (HG2) and red (HG3) and correspond to the haplogroups labeled on the maximum likelihood tree (Figs. 3 and 4). The overall haplotype diversity for all samples was 0.610, Tajima’s D was 1.99 (ns, *P* > 0.05), and the overall nucleotide diversity was 0.016 (Table 5).

**Table 5:** Haplotype diversity, nucleotide diversity and Tajima’s D for each of the six regions included from El Salvador

	Population	No.of haplotypes	Haplotype diversity	Nucleotide diversity	Tajima’s D
1	Sonsonate	2	0.167	0.003	-2.043*
2	San Salvador	4	0.764	0.017	1.400 ns
3	Chalatenango	4	0.745	0.009	1.417 ns
4	Usulután	3	0.538	0.008	1.697 ns
5	San Miguel	6	0.773	0.008	0.678 ns
6	Morazán	7	0.774	0.011	1.454 ns
	Overall	10	0.610	0.015	1.990 ns

*Abbreviation*: *ns* not significant at *P* < 0.05

*P < 0.05

Analyses of haplotype diversity, nucleotide diversity and Tajima’s D were also determined for each of the 6 regions (Table 5). Sonsonate on the Pacific coast had the smallest number of haplotypes (2) and lowest nucleotide diversity (0.003). Tajima’s D was only significant for Sonsonate (-2.043, *P* < 0.01) and not for the other five regions (*P* > 0.10, Table 5). San Salvador (the capital) had 4 haplotypes, a haplotype diversity of 0.764 and a nucleotide diversity of 0.017, while Morazán had 7 haplotypes, a haplotype diversity of 0.774 and nucleotide diversity of 0.011 (Table 5). The three haplogroups found mtDNA sequences varied in the number of haplotypes, haplotype diversity and nucleotide diversity. The first haplogroup (indicated in blue) had 2 haplotypes, a haplotype diversity of 0.041 and a nucleotide diversity of 0.00014, and the lowest genetic diversity of the three haplogroups (Fig. 4). The second haplogroup (indicated in green) had 4 haplotypes, a haplotype diversity of 0.577, and a nucleotide diversity of 0.002. The third haplogroup (indicated in red) consisted of 4 haplotypes, and had the highest haplotype and nucleotide diversity, 0.682 and 0.0075, respectively (Fig. 4).

**Fig. 5:**
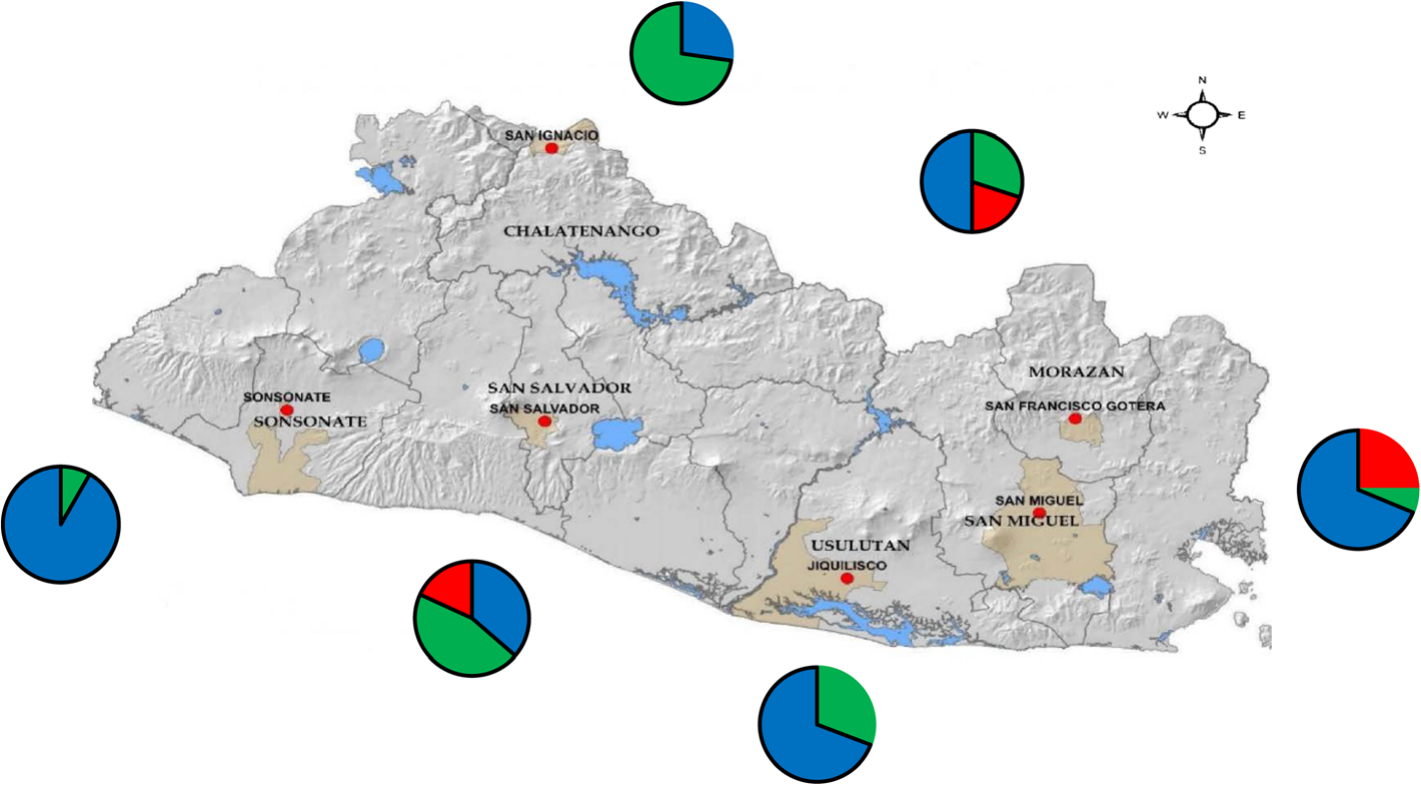
Map of collection locations showing the proportion of each haplogroup in the mtDNA sequences from each region

For each of the six regions, the proportion of samples belonging to each haplogroup was found and mapped onto its corresponding region of the country (Fig. 5). For Sonsonate, the majority of sequences were in haplogroup 1 (blue) (Fig. 5). San Salvador had a mix of the three haplogroups. Of the six regions sampled, Chalatenango had the largest number of sequences in haplogroup 2 (73%) (green). Jiquilisco, Usulután 69% of samples in haplogroup 1 (blue) and 31% in haplogroup 2. San Miguel and Morazán samples had the largest number of sequences which belonged to haplogroup 3, shown in red (Fig. 5). San Miguel had 69% of samples in haplogroup 1 (blue), and 25% in haplogroup 3 (red, Fig. 5), while Morazán had ~50% of samples in haplogroup 1, 30% in haplogroup 2 and 20% in haplogroup 3 (red, Fig. 5).

**Fig. 6:**
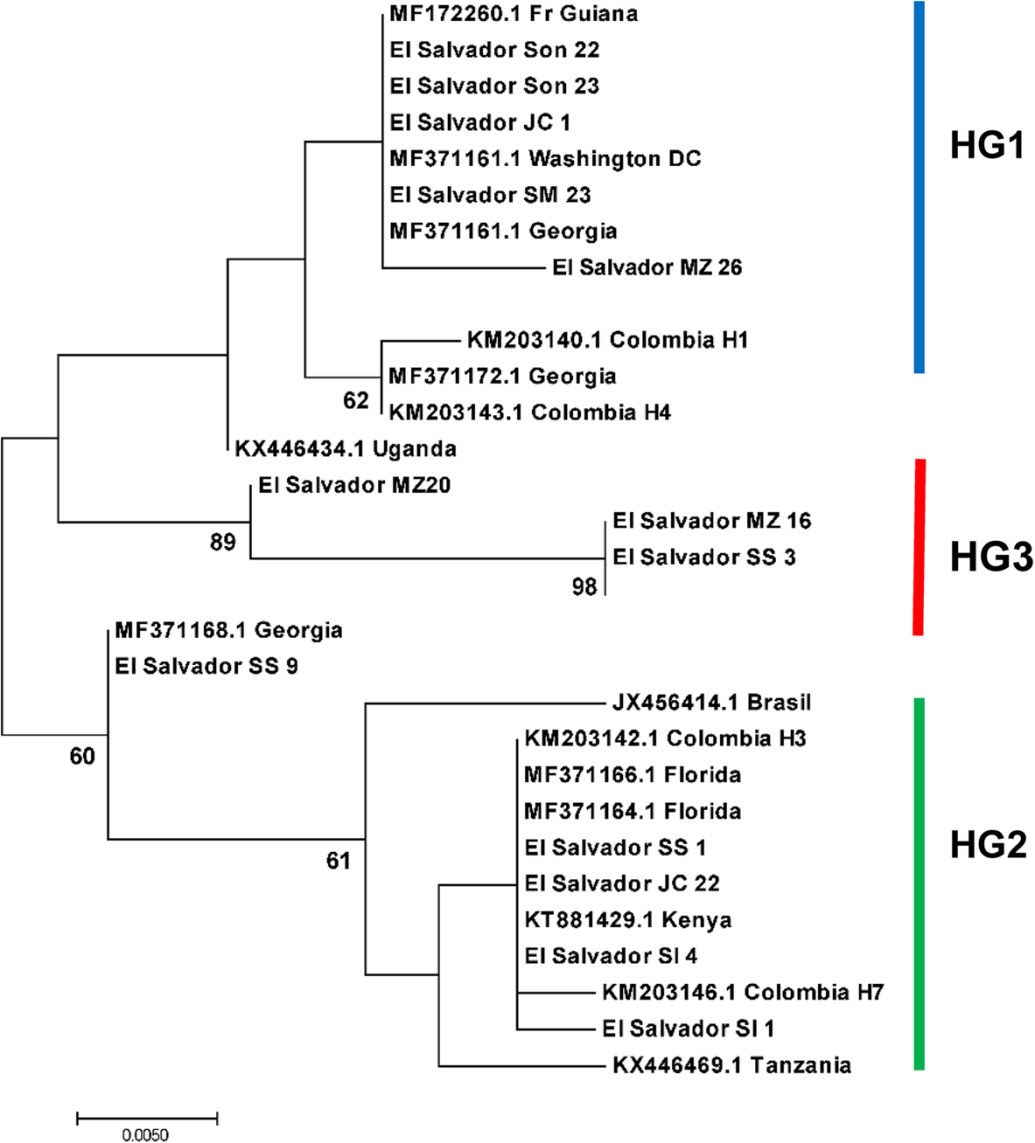
Maximum likelihood tree based on the H-K-Y model, of samples from each region of El Salvador and each haplogroup, combined with *Ae. aegypti* sequences from GenBank. *Abbreviations*: Son, Sonsonate; SS, San Salvador; SI, San Ignacio, Chalatenango; JC, Jiquilisco, Usulután; SM, San Miguel; Mz, Morazán

The combined phylogenetic tree consisted of 14 individuals from El Salvador and 16 sequences from GenBank produced a tree with two main lineages (Fig. 6). The first lineage contained El Salvador samples from haplogroup 1 (JC 1, Son 23, SM 23 and Son 22) clustered with GenBank accessions MF172260 from French Guyana, MF 371161 Washington DC, USA, KM203140.1 Colombia H1, KM203143.1 Colombia H4 and MF371161.1 Georgia, USA. This lineage also had a subtree that consisted of three individuals from haplogroup 3 (red) from El Salvador (SS 3, MZ 16, SM 4) (Fig. 6). The second major lineage in the combined maximum likelihood tree consisted of samples from El Salvador haplogroup 2 (green) from this study (JC 22, SS 1, SI 4, SI 1, SS 9) which grouped together with GenBank accessions MF371164 from Florida USA, KM203146 Colombia H7, KM203142.2 Colombia H3, and JX456414.1 Brazil, among others. A subtree contained MF371168.1 from Georgia, which grouped closely with samples from El Salvador SS 9 (Fig. 6).

## Discussion

In this study, two molecular markers were used to examine the genetic diversity of *Ae. aegypti* in El Salvador, Central America. Data from this study found a high level of genetic diversity among samples and suggest at least two lineages have been introduced to El Salvador. The previous eradication program of *Ae. aegypti* in much of the Western Hemisphere was followed by a reemergence of this insect in most of its previous range, including in El Salvador. This may be due to the ease of movement of insect eggs and increased trade and migration. Few data from Central America on the genetic diversity of *Ae. aegypti* were available for comparison, but samples were compared with others from North and South America to consider the source of the introductions.

The data from the Structure Harvester analysis of the AFLP data found there were two genetically divergent groups of *Ae. aegypti* in El Salvador. The northern region of the country including Sonsonate, San Salvador and Chalatenango consisted of one genetically distinct group, while individuals from San Miguel in the eastern and interior region of the country, consisted of a second genetically divergent group. Samples from Jiquilisco (Usulután) and Morazán, also in the south and eastern region, consisted of a mix of the two groups. Genetic distances were largest between San Miguel and Chalatenango (nearly 10%) and the overall AMOVA of the six populations found 11% genetic variation. These values are similar to those found in other studies; for example, 15% in Colombia [47], 19% in Argentina [12] and 20% in Mexico [11]. The principal components analysis found that the six populations located in three quadrants, and isolation by distance was significant. The largest variation was explained by the first axis (44.75%), with the departments in the north and west (Chalatenango, San Salvador, Sonsonate) separating from those in the southeast (Jiquiliscco, San Miguel and Morazan). The combined results from the PCA analysis and the Structure Harvester analysis suggest that individuals from the northwest region of the country, Chalatenango, San Salvador and Sonsonate, have some degree of genetic isolation from the three populations in the south.

There are several explanations for the two genetically distinct groups found in the Structure Harvester analysis. Studies have suggested at least two subspecies of *Ae. aegypti* [3, 7, 48] which occur in different habitats. However, in this study, larvae were collected from the same type of habitat, from barrels and wash basins (‘pilas’) outside homes in neighborhoods, and all individuals in this study are believed to be *Ae. aegypti aegypti*. Other studies have suggested that divergence may also occur between populations from the rainy season and the dry season [9]. Seasonal divergence is not likely to explain the genetic divergence observed in this study, since all collections were made from May to August during the rainy season. Rather, the genetic divergence appears to follow a spatial pattern along a northwest to southeast gradient.

A large degree of genetic diversity was also found in the mitochondrial DNA sequences in this study. There were three haplogroups with a total of 10 haplotypes, with an overall haplotype diversity of 0.610 and a nucleotide diversity of 0.015. These values are relatively high, and are similar to those found by Bennett et al. [6] for samples from America (11 haplotypes and a nucleotide diversity of 0.013). Samples from El Salvador also had a nucleotide diversity similar to that found in studies for *Ae. aegypti* in Colombia and Venezuela [47, 49]. In El Salvador, the areas with the most haplotypes occurring were Morazán and San Miguel. These regions are close to the Gulf of Fonseca, which borders El Salvador, Honduras and Nicaragua on the Pacific coast. The higher genetic diversity in this region may represent introduced populations from regions which were not subject to eradication programs.

The most abundant haplotype sequenced in the present study was haplotype 1, which was found in all six regions of the country. In Sonsonate, on the Pacific coast of El Salvador, over 90% of individuals sequenced were haplogroup 1, and the nucleotide diversity in Sonsonate was the lowest of all regions (0.003) (Fig. 5). Low nucleotide diversity could indicate a bottleneck after a small number of individuals were introduced, or perhaps a population which was reduced in size due to insecticides [11]; these hypotheses require testing. The Tajima’s D value for Sonsonate was significant (-2.043), suggesting a population expansion in this region. This haplogroup was the most widespread, suggesting it has been in El Salvador longer than the other two haplogroups. It may have been moved into other departments by passive transport [50].

In El Salvador, the second most abundant haplotype observed (h2, green) occurred more frequently in San Ignacio, Chalatenango than in the other five departments (Fig. 5). San Ignacio is a cool (~25 °C), high elevation (~2500 m above sea level) mountainous area on the northeastern border of El Salvador and Honduras. San Ignacio, Chalatenango had four haplotypes, a haplotype diversity of 0.577 and a nucleotide diversity of 0.009. The bottleneck test found this group also had a significant departure from an equilibrium of heterozygotes, indicating a recent population bottleneck. Individuals in this haplogroup clustered in the phylogenetic tree (Fig. 6) with those from Brazil and Florida, as well as Colombia; the combined maximum likelihood tree had two main lineages with low to moderate bootstrap support. Previous studies of *Ae. aegypti* have similarly found phylogentic trees with two lineages and varying levels of support [44, 48, 49]. Haplogroup two may have been introduced in El Salvador in the post-eradication era; it has a higher nucleotide and haplotype diversity than haplogroup 1, and a more limited distribution. However, further work would be needed to test this hypothesis.

A third haplogroup (red, Fig. 6) was found in the mtDNA *cox*1 sequences from El Salvador. These individuals formed a small subgroup of the first lineage of the combined phylogenetic tree. While several previous studies of *Ae. aegypti* have suggested that three lineages have been introduced in the Western Hemisphere [12, 15], more recent studies and this one suggest that two divergent groups of *Ae. aegypti* have been introduced into El Salvador. The third haplogroup in our study was found in the capital city San Salvador, and in the south and east portion of El Salvador, in San Miguel and Morazán. The third (*red*) haplogroup clustered in the first lineage with the blue haplogroup from El Salvador, yet had much higher haplotype and nucleotide diversity. This group had no match to sequences in GenBank which was 99–100% similar, suggesting it is from an area which has yet to be included in global level population analyses.

## Conclusions

This study of *Ae. aegypti* genetic variability in El Salvador agrees with other studies in the Western Hemisphere in finding at that at least two genetically divergent groups have been introduced. Introductions may have occurred through ports with international cargo or *via* transport along major corridors such as the Pan American Highway between adjacent Central American countries. The genetic variability of the populations of *Ae. aegypti* in Central America has only recently been investigated. Future work should expand this study to examine genetic diversity of *Ae. aegypti* in surrounding countries which will contribute to our understanding of the reintroductions of this invasive species in Central America.

## Additional files

Additional file 1: **Figure S1.** Results from a Structure Harvester analysis of all six populations of *Ae. aegypti*. Structure Harvester uses the results from Structure to calculate the Delta K value, the change in likelihood, for the number of potential clusters. Structure Harvester calculated the most likely number of clusters was 2 (K = 2) (TIFF 4202 kb)

Additional file 2: **Figure S2.** Results from a Structure Harvester analysis of all six populations of *Ae. aegypti* in this study. Each row shows the probability of K populations and delta K. The most likely number of populations was K = 2. (TIFF 5816 kb)

Additional file 3: **Figure S3.** Mantel test to examine the relationship between genetic distance and geographical distance for six populations of *Ae. aegypti* (*r* = 0.976, *P* = 0.010). (TIFF 1791 kb)

Additional file 4: **Figure S4.** Principal components analysis using genetic distance output of AMOVA of the six populations of *Ae. aegypti* in the study. (TIFF 1744 kb)
